# Ceramide and C1P: a lipid love story of *Brassica*-*Sclerotinia* interaction

**DOI:** 10.1093/plphys/kiae656

**Published:** 2024-12-17

**Authors:** Ritu Singh, Prem Pratap Singh

**Affiliations:** Assistant Features Editor, Plant Physiology, American Society of Plant Biologists; Department of Plant Science, University of California, Davis, CA 95616, USA; Department of Viticulture & Enology, University of California, Davis, CA 95616, USA

Lipids are essential cellular constituents in plants that fulfill diverse roles in structural integrity, energy storage, signal transduction, and defense mechanisms (Kuźniak and Gajewska 2024). Among these lipids, sphingolipids, particularly ceramides, have emerged as critical players in plant immunity, orchestrating responses to environmental stress and pathogen attacks (Liu et al. 2021). Ceramides are central bioactive molecules in sphingolipid metabolism that influence fundamental processes such as cell differentiation, apoptosis, and immune regulation across eukaryotic systems.

In plants, ceramides and their phosphorylated derivatives, ceramide-1-phosphate (C1P), play dual roles in programmed cell death (PCD), a key defense strategy against pathogens (DeMell et al. 2023). *Ceramide kinase* (*CERK*) phosphorylates ceramides at the C1 position, converting them to C1Ps, and this phosphorylation is crucial for maintaining the balance between ceramide and C1P (Hannun and Obeid 2008). While ceramide accumulation is associated with the activation of PCD, C1P can counteract ceramide-induced cell death (Liang et al. 2003). However, the mechanisms by which C1P contributes to plant defense remain poorly understood, underscoring the need to explore the role of CIP and C1P-binding proteins in disease resistance.

*Brassica napus* (rapeseed or canola) is a globally important oilseed crop that provides edible oil, biodiesel, and protein-rich animal feed, contributing significantly to agricultural economies (Taylor et al. 2018). However, the cultivation of this vital crop is threatened by *Sclerotinia sclerotiorum*, a necrotrophic fungal pathogen that infects over 400 plant species. A recent study published in *Plant Physiology* by Ouyang et al. (2024) sheds light on the sphingolipid metabolism gene *BnaCERK* and reveals its role in enhancing *B. napus* resistance to *S. sclerotiorum*.

Ouyang and colleagues inoculated *B. napus* leaves with *S. sclerotiorum* and tracked changes over 10 hours post-inoculation. By 10  hours post inoculation, microscopic necrotic spots and visible leaf wrinkling were observed. Sphingolipid profiling revealed a decline in ceramide levels accompanied by a rise in C1P levels. Transcriptomic analysis showed that upregulated differentially expressed genes were enriched in pathways related to the plasma membrane, protein kinase activity, and transcription factors, whereas downregulated genes were associated with the plasma membrane and lipid transport pathways. Notably, among sphingolipid metabolism genes, *BnaCERK* exhibited a 3.33-fold upregulation post infection, indicating its role during pathogen attack.

Further, to assess the functional role of *BnaCERK* in pathogenesis, the authors generated transgenic *B. napus* plants overexpressing (OE) *BnaCERK* and used CRISPR/Cas9 to knock out *BnaCERK* homologs in the A and C subgenomes. Ceramide levels were significantly higher in knockout lines compared with wild-type (WT) and OE plants, whereas OE plants exhibited a marked reduction in ceramide and an increase in C1P level, as well as enhanced resistance to the pathogen. Consistent with the known effect of ceramide in promoting PCD, knockout lines displayed significantly larger necrotic lesion areas than WT, while OE lines exhibited smaller lesion areas. Moreover, knockout lines showed increased salicylic acid levels compared with WT and OE plants, while jasmonic acid levels were significantly elevated in OE lines. Reactive oxygen species burst triggered by flg22 was higher in knockout lines compared with WT and OE lines, suggesting altered defense signaling (Fig. 1).

**Figure 1.**
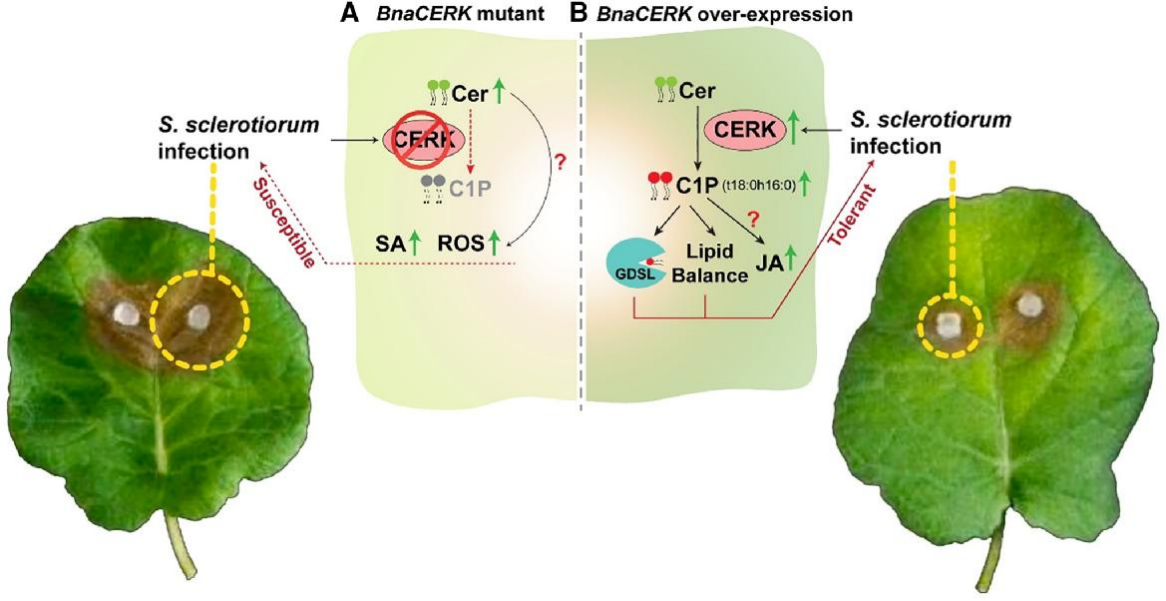
Proposed model of CERK- and C1P-mediated regulation in the *S. sclerotiorum–B. napus* interaction (adapted from Ouyang et al. (2024) figures 4A and 7G). **A)** Susceptible plant (*BnaCERK* mutant): In plants lacking the BnaCERK enzyme, ceramide (Cer) cannot be converted into ceramide-1-phosphate (C1P). As a result, ceramide levels accumulate in the cells, leading to increased reactive oxygen species (ROS) production and elevated salicylic acid (SA) levels. The exact pathway linking ceramide to these changes remains unknown. This imbalance renders the plant more susceptible to *S. sclerotiorum* infection. **B)** Resistant/Tolerant plant (*BnaCERK* overexpression): *S. sclerotiorum* infection triggers upregulation of *CERK*, which phosphorylates ceramide into C1P. C1P binds to the GDSL enzyme, potentially enhancing its activity. Additionally, jasmonic acid (JA) levels are elevated in these plants, although the underlying mechanism is unclear. The combined effect of these responses contributes to plant resistance against *S. sclerotiorum*. The “?” indicates an unidentified pathway and the green arrow represents upregulation.

The authors hypothesized that C1P interacts with downstream effector proteins to mediate defense responses. Using lipid-coated beads, 172 potential C1P-binding proteins were identified, 43 of which were enriched in biotic stimulus pathways. Among these, the *BnaGDSL* gene, previously linked to *S*. *sclerotiorum* resistance (Ding et al. 2020), was induced post infection. Structural modeling predicted that C1P, through its phosphate head group, interacts with BnaGDSL. The interaction between C1P and BnaGDSL was further confirmed through liposome assays and surface plasmon resonance. To understand whether C1P influences the enzymatic activity of BnaGDSL, recombinant GDSL protein was incubated with 2 lipid substrates: p-nitrophenyl butyrate and p-nitrophenyl acetate. The results demonstrated that BnaGDSL efficiently hydrolyzed these substrates compared with the GST control, indicating that BnaGDSL has lipase activity. Furthermore, the enzymatic activity of BnaGDSL increased with higher C1P concentrations, suggesting that C1P binding enhances BnaGDSL activity. These results suggest that C1P enhances *BnaGDSL* activity, bolstering plant resistance against *S. sclerotiorum*.

This study highlights the role of sphingolipid metabolism in plant defense during *S*. *sclerotiorum* infection. The conversion of ceramide to C1P during pathogen attack is crucial for regulating plant immunity (Fig. 1). Overexpression of the *BnaCERK* gene increases C1P levels, causing increased resistance to *S*. *sclerotiorum*, while elevated ceramide levels in knockout plants result in greater susceptibility. The balance between ceramide and C1P is key in determining plant resistance, with C1P not only playing a role in modulating defense signaling but also enhancing the activity of the GDSL enzyme. Moreover, the interaction between C1P and *BnaGDSL* is critical in mitigating necrotrophic fungal infections, underscoring the importance of sphingolipid-mediated defense pathways. In conclusion, this research provides valuable insights into how C1P enhances resistance against necrotrophic fungal pathogens and lays the groundwork for innovative strategies to improve crop protection through sphingolipid regulation.

## Data Availability

No data is generated in this study.
